# Simultaneous UPLC–TQ-MS/MS determination of six active components in rat plasma: application in the pharmacokinetic study of *Cyclocarya paliurus* leaves

**DOI:** 10.1186/s13020-019-0248-7

**Published:** 2019-08-06

**Authors:** Zi-Wan Ning, Li-xiang Zhai, Jiao Peng, Ling Zhao, Tao Huang, Cheng-yuan Lin, Wei-hong Chen, Zhen Luo, Hai-tao Xiao, Zhao-xiang Bian

**Affiliations:** 1 Clinical Division, School of Chinese Medicine, Hong Kong Baptist University, Baptist University Road 7, Kowloon, Hong Kong SAR China; 2 School of Pharmaceutical Sciences, Health Science Center, Shenzhen University, Shenzhen, 518060 China; 3 Department of Pharmacy, Peking University Shenzhen Hospital, 518035 Shenzhen, China; 4 Infinite Chinese Herbal Immunity Research Center, Tianhe District, Guangzhou, 510000 China; 5 School of Food Science and Engineering, South China University of Technology, Panyu District, Guangzhou, 510006 China

**Keywords:** *Cyclocarya paliurus*, UPLC–TQ-MS/MS, Simultaneous determination, Pharmacokinetics

## Abstract

**Background:**

*Cyclocarya paliurus* (Batal.) Ijinskaja (CP) is a monotypic genus plant, also called sweet tea tree that belongs to the Juglandaceae family, which is mainly distributed in the subtropical highlands in China. Our previous work has verified that CP leaves exhibit a potent hyperglycemic effect by inhibiting pancreatic β cell apoptosis through the regulation of MPAK and Akt signaling pathways. However, the components that contribute to this potential health benefit remain undiscovered.

**Method:**

A sensitive, reliable, and validated ultra-performance liquid chromatography coupled with triple-quadrupole tandem mass spectrometry (UPLC–TQ-MS/MS) method was developed to simultaneously determine the presence of six active components (neochlorogenic acid, chlorogenic acid, quercetin-3-*O*-glucuronide, kaempferol-3-*O*-rhamnoside, quercetin, and kaempferol) in rat plasma after a single oral administration (in a dosage of 10.5 g/kg) of an extract of CP leaves to rats. The separation was performed on a Waters ACQUITY BEH C_18_ column (50 mm × 2.1 mm, 1.7 μm). The detection was conducted by multiple reaction monitoring (MRM) in negative ionization mode. The two highest abundant MRM transitions without interference were optimized for each analyte. Acetonitrile and formic acid aqueous solution (0.1%) was used as the mobile phase at a flow rate of 0.3 ml/min.

**Result:**

The precision, accuracy, and recovery all satisfied the criteria of international guidance (Bioanalytical Method Validation Guidance for Industry, Food and Drug Administration), and the analytes were stable in plasma for all tested conditions. The main pharmacokinetic parameters were calculated by plasma concentration versus time profiles using the pharmacokinetics program.

**Conclusion:**

The pharmacokinetic parameters of each compound can facilitate future clinical studies.

## Background

*Cyclocarya paliurus* (Batal.) Ijinskaja (CP) is a monotypic genus plant that belongs to the Juglandaceae family, which is mainly distributed in the subtropical highlands in China [1]. CP leaves have long been used as a traditional Chinese medicinal herb, as they have heat- and toxin-clearing attributes and are used to treat obesity and diabetes; the leaves have also been historically consumed as nutraceutical tea [2]. In the previous decade, a large number of modern chemical investigations have indicated that CP leaves contain hundreds of compounds, such as phenolic acids, flavonoids, and triterpenoids [3]. Moreover, our previous work has verified that CP leaves have a potent hyperglycemic effect by inhibiting pancreatic β cell apoptosis through the regulation of MPAK and Akt signaling pathways [4]. However, the components that contribute to this potential health benefit remain undiscovered.

According to serum pharmacochemistry, only the components absorbed into the blood will have the opportunity to exert pharmacological bioactivities [5]. We analyzed the absorbed components of the CP extract in rat plasma by using UPLC-Q-TOF/MS; 13 absorbed components were identified—quinic acid, gallic acid, neochlorogenic acid, chlorogenic acid, *p*-hydroxybenzoid acid, quercetin-3-*O*-glucuronide, kaempferol-3-*O*-glucopyranoside, kaempferol-3-*O*-rhamnoside, quercetin, kaempferol, quadranoside IV, asiatic acid-, and loganin-7-*O*-pentoside [1]. These compounds can be classified into three groups—organic acids, flavonoids, and triterpenes; some of these compounds have been reported to have potent beneficial effects in the treatment of diabetes. Among organic acid compounds, for example, chlorogenic acid was reported to effectively prevent diabetic nephropathy by inhibiting oxidative stress and inflammation through the modulation of the Nrf2/HO-1 and NF-ĸB pathways [6]. In addition, neochlorogenic acid possesses significant inhibitory activity against rat lens aldose reductase and advanced glycation end products [7]. Further, gallic acid was found to effectively improve the glucose uptake of insulin-resistant mouse hepatocytes and decrease hyperglycemia and hepatic glucose metabolism of diabetic rats on a high-fructose diet [8]. Among flavonoids, various studies have indicated that both quercetin and kaempferol exert potential antidiabetic effects in regulating insulin secretion [9, 10], controlling insulin resistance [11, 12], and reducing glucose absorption [13]. Moreover, flavonoid glucosides—such as quercetin-3-*O*-glucuronide, kaempferol-3-*O*-glucopyranoside, and kaempferol-3-*O*-rhamnoside—have also played an essential role in treating diabetes. It has been reported that quercetin-3-*O*-glucuronide is as effective as quercetin in ameliorating insulin resistance by regulating the IRS-1 function of endothelium [14]; kaempferol-3-*O*-rhamnoside can significantly stimulate GLUT-4 translocation and synthesis in adipocytes [15]; and kaempferol-3-*O*-glucopyranoside possesses potent α-glucosidase inhibitory activity [16, 17]. Loganin-7-*O*-pentoside is a flavonoid glucoside of loganin, which has been reported to effectively decrease the fasting blood glucose levels in diabetic mice [18]. Among triterpenes, asiatic acid—a triterpenoid isolated from the CP extract—was also disclosed to effectively improve glucose homeostasis of skeletal muscle by increasing the expression of GLUT4 [19]. These findings imply that the abovementioned active compounds may be responsible for the anti-diabetic effects of the CP extract. With respect to establishing its efficacy in treating diabetes, it is necessary to investigate the pharmacokinetic behavior of multiple active compounds in the extract of CP leaves. Thus far, no reports are available on the simultaneous determination and pharmacokinetic studies of the abovementioned main active components in the CP extract. Among these compounds, quinic acid, gallic acid, kaempferol-3-*O*-glucopyranoside, asiatic acid, and loganin-7-*O*-pentoside were found in rather low concentrations in the CP extract; in contrast, neochlorogenic acid, chlorogenic acid, quercetin-3-*O*-glucuronide, kaempferol-3-*O*-rhamnoside, quercetin, and kaempferol are the main compounds with high concentration in the CP extract [4]. In this study, a sensitive and reliable ultra-high-performance liquid chromatography coupled with a triple quadrupole electrospray tandem mass spectrometry (UPLC–TQ-MS/MS) with the multiple reactions monitoring (MRM) method was developed to simultaneously quantify the abovementioned six main active components in rat plasma after oral administration of the CP extract. The results of this study would be helpful for improving clinical therapeutic efficacy and for further pharmacological studies on CP leaves.

## Methods

### Regents and materials

**Fig. 1:**
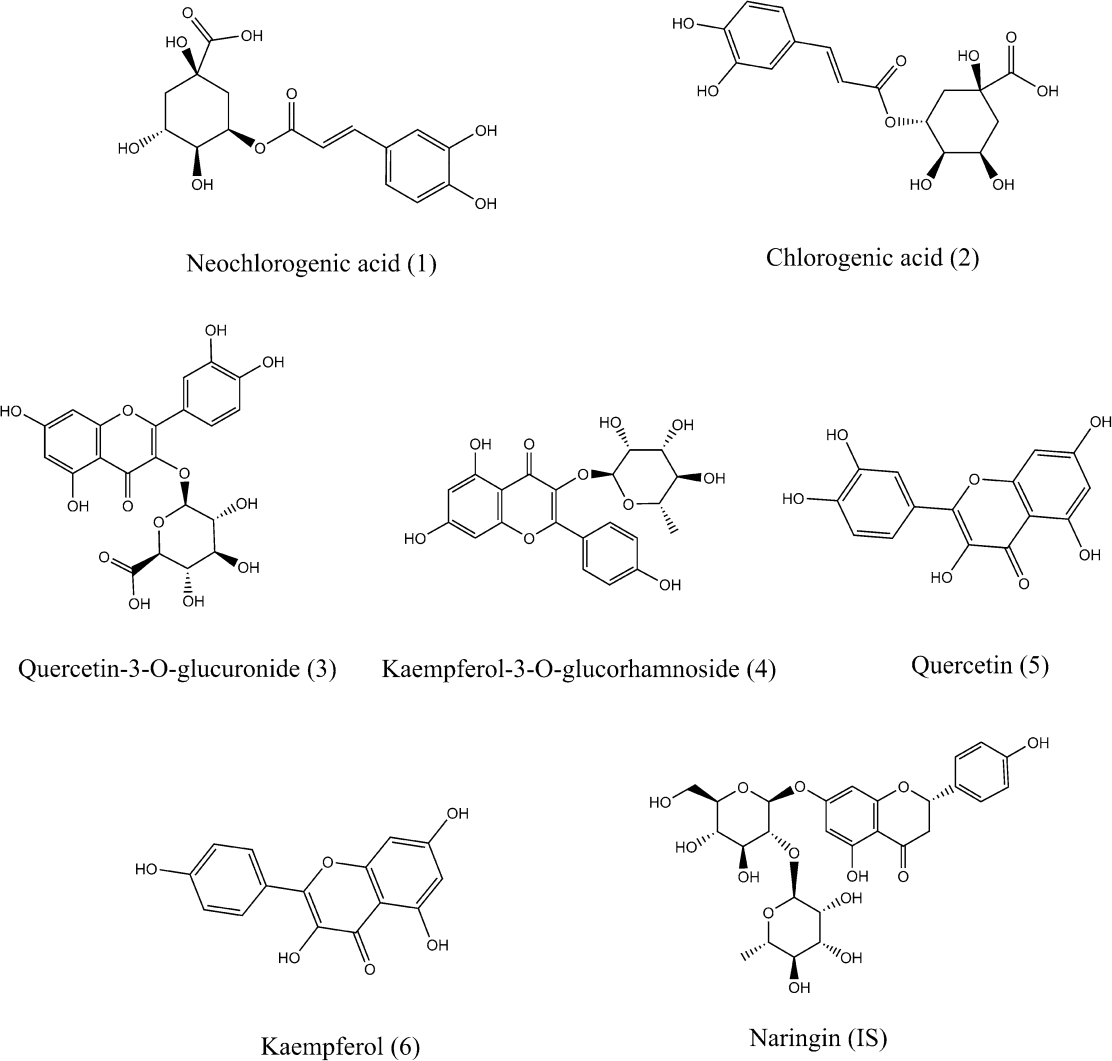
Chemical structure of six components. Neochlorogenic acid (1), chlorogenic acid (2), quercetin-3-*O*-glucuronide (3), kaempferol-3-*O*-rhamnoside (4), quercetin (5), kaempferol (6), and naringin (IS)

Reference standards of neochlorogenic acid (**1**, Purity ≥ 98%), chlorogenic acid (**2**, Purity ≥ 98%), quercetin-3-*O*-glucuronide (**3**, Purity ≥ 98%), kaempferol-3-*O*-rhamnoside (**4**, Purity ≥ 98%), quercetin (**5**, Purity ≥ 98%), and kaempferol (**6**, Purity ≥ 98%) were supplied by Baoji Herbest Bio-Tech Co., Ltd. (Shanxi, China). Naringin (**7**, internal standard (IS)) was purchased from Shanghai Yuanye Bio-Technology Co., Ltd (Shanghai, China). Their chemical structures are illustrated in Fig. 1. Acetonitrile, methanol, and formic acid (HPLC grade) were purchased from Merck (Darmstadt, Germany). Ultra-pure water was purified by the Millipore water purification system (Millipore, Milford, MA, United States).

### Ethical statement

Six male Sprague–Dawley rats (220–250 g) were purchased from the Experimental Animal Center, The Chinese University of Hong Kong (Hong Kong, China). The rats were bred in an environmentally controlled room (22 ± 2 °C, relative humidity 50 ± 20%) with a natural light–dark cycle for 7 days before the experiment was conducted. The study protocol was approved by the Ethical Committee of Hong Kong Baptist University in accordance with “Institutional Guidelines and Animal Ordinance” of the Department of Health, Hong Kong Special Administrative Region (Registration No. LIUYE/15-16/01-CLNC).

### Preparation of the CP methanol extract

The crude CP extract was prepared according to previous protocol [4]. For preparation of the CP extract, air-dried CP leaves (5 kg) were boiled in water (60 l water for the first time and 50 l water for the second time) twice (boiled for 2 h the first time and for 1 h the second time). Thereafter, the extract was concentrated and dried under reduced pressure to yield the crude extract (830 g), and then the crude extract was lyophilized and stored at 4 °C in the refrigerator until use.

### Chromatography and mass spectrometry conditions

Liquid chromatographic analysis was performed on an Agilent 1290 ultra-performance liquid chromatography system, comprising a 1290 binary pump solvent management system, a 1290 TCC, and an 1290 auto-sampler. A Waters ACQUITY BEH C_18_ column (50 mm × 2.1 mm, 1.7 μm) was employed for the separation of samples, and the column temperature was maintained at 40 °C. The mobile phase comprised A (0.1% formic acid) and B (0.1% formic acid in acetonitrile), using a gradient elution of 2–5% B for 0–1 min, 5–40% B for 1–5 min, 40–75% B for 5–8 min, 75–100% B for 8–8.5 min, 100% B for 8.5–11 min and then returned to the initial condition with the flow rate set at 0.30 ml/min. The auto-sampler was conditioned at 4 °C, and the injection volume was 2 μl.

Mass spectrometry detection was performed using an Agilent 6460 Triple Quadrupole MS equipped with an Agilent Jet Stream electrospray ionization source (ESI). The ESI source was set in negative ionization mode. The parameters in the source were set in the following manner: capillary voltage, 3.5 kV; source temperature, 150 °C drying gas temperature, 300 °C drying gas flow, 8 l/min; nebulizer, 45 psi; sheath gas temperature, 350 °C sheath gas flow, 8 l/min. Analyte detection was performed using MRM. For each analyte, the two highest abundant MRM transitions without interference in sample were selected—one for quantification and the other for confirmation. The fragment or voltage and collision energy were optimized for precursor/product ion pairs of each analyte; the selected values are presented in Table 1.

**Table 1:** Precursor/product ion pairs and parameters for MRM of compounds used in this study

Analyte	RT (min)	Precursor ion species	MRM transition		Frag. (V)	CE (eV)	Dwell time (ms)	Ionization mode
			Precursor ion → Product ion (*m/z*)					
Neochlorogenic acid	1.97	(M−H)^−^	353.1 → 191.0	Quantifier	104	9	20	Negative
			353.1 → 135.0	Qualifier	104	29	20	
Chlorogenic acid	2.45	(M−H)^−^	353.1 → 191.0	Quantifier	104	9	20	Negative
			353.1 → 135.0	Qualifier	104	29	20	
Quercetin-3-*O*-glucuronide	3.44	(M−H)^−^	477.1 → 301.1	Quantifier	110	17	20	Negative
			477.1 → 151.0	Qualifier	110	37	20	
Kaempferol-3-*O*-rhamnoside	4.04	(M−H)^−^	431.1 → 285.0	Quantifier	170	13	20	Negative
			431.1 → 255.0	Qualifier	170	37	20	
Quercetin	4.50	(M−H)^−^	301.0 → 151.0	Quantifier	114	17	20	Negative
			301.0 → 179.0	Qualifier	114	9	20	
Kaempferol	5.03	(M−H)^−^	285.0 → 185.1	Quantifier	170	27	20	Negative
			285.0 → 117.0	Qualifier	170	43	20	
Naringin (I.S.)	3.80	(M−H)^−^	579.2 → 271.0	Quantifier	150	15	20	Negative
			579.2 → 151.0	Qualifier	150	30	20	

### Preparation of calibration standards and the quality control (QC) sample

The stock solutions of neochlorogenic acid (150 μg/ml), chlorogenic acid (150 μg/ml), quercetin-3-*O*-glucuronide (100 μg/ml), kaempferol-3-*O*-rhamnoside (20 μg/ml), quercetin (1 μg/ml), kaempferol (5 μg/ml), and IS (20 μg/ml) were prepared in methanol, respectively. Then, the series of working solutions were obtained by further dilution with methanol.

Using the same method as that for the calibration samples, low, middle, and high concentrations of quality control (QC) samples were independently prepared at concentrations of 120 ng/ml, 1200 ng/ml, and 12,000 ng/ml for neochlorogenic acid; 120 ng/ml, 1200 ng/ml, and 12,000 ng/ml for chlorogenic acid; 40 ng/ml, 400 ng/ml, and 4000 ng/ml for quercetin-3-*O*-glucuronide; 5 ng/ml, 50, ng/ml, and 500 ng/ml for kaempferol-3-*O*-rhamnoside; 1.5 ng/ml, 15 ng/ml, and 150 ng/ml for quercetin; and 20 mg/ml, 200 ng/ml, and 2000 ng/ml for kaempferol. All stock solutions and working solutions were stored at − 20 °C until use.

### Plasma sample preparation

Plasma samples were thawed at room temperature before analysis. Then, 10 μl of IS solution (20 μg/ml) and 200 μl of methanol were added to the plasma sample (90 μl) in a 1.5 ml Eppendorf tube. The mixture was vortexed for 1 min and centrifuged at 12,000×*g* for 10 min at 4 °C to deproteinize. Thereafter, 2 μl of the supernatant was injected into the UPLC–TQ/MS system for analysis.

### Method validation

#### Specificity

The specificity was calculated by comparing blank plasma samples, blank plasma samples spiked with standards and internal standards, and plasma samples collected after oral administration of the CP extract and spiked with IS.

#### Linearity and lower limit of quantification (LLOQ)

For the calibration curve, the mixture stock solution was diluted with methanol to make a series of working solutions. The calibration samples were prepared independently by adding a series of working solutions with different concentrations (10 μl), IS solution (10 μl), and 190 μl methanol to blank rat plasma (90 μl) to determine linearity and the lower limit of quantification (LLOQ).

#### Precision and accuracy

The precision and accuracy of the method were evaluated by analysis of the three QC samples. The interday and intraday precision was determined using low, middle, and high concentrations which were mentioned in “Preparation of calibration standards and the quality control (QC) sample”. The precision was expressed by relative standard deviation (RSD %), and accuracy was expressed by relative error (RE %).

#### Recovery and matrix effects

The extraction recoveries of analytes were determined by comparing the peak areas of the QC samples pre-spiked in blank plasma with those post-spiked in blank plasma (n = 3). In addition, the matrix effect was determined by comparing the peak areas of the QC samples pre-spiked in blank plasma with those in the solvent (n = 3).

#### Stability

The stability of analytes in the plasma was determined by using the QC samples under three conditions: (1) short-term stability—QC samples (n = 3) were stored at room temperature for 24 h and refrigerated (4 °C) for 24 h; (2) long-term stability—QC samples (n = 3) were stored at − 20 °C for 15 days; (3) three freeze–thaw cycles stability—QC samples (n = 3) were detected after three cycles of freezing (− 20 °C) and thawing (ambient temperature).

### Pharmacokinetics study

Six healthy male Sprague–Dawley rats were fasted for 12 h with free access to water prior to the experiment. The CP extract was administered to rats by oral gavage at a dose of 10.5 g/kg body weight. Blood samples of approximately 0.5 ml were collected at 15 and 30 min and at 1 h, 2 h, 3 h, 4 h, 6 h, 8 h, 10 h, 12 h, and 24 h in heparinized centrifuge tubes from the ophthalmic vein using a sterile capillary tube under anesthesia after oral administration of the CP extract. Following centrifugation at 4000×*g* for 10 min at 4 °C, plasma samples were transferred to polypropylene tubes and stored at − 80 °C until analysis.

The concentrations of six analytes in plasma at different time points were expressed as mean ± SD, which was calculated from the daily calibration curve. All the pharmacokinetic parameters were processed through non-compartmental analysis using the DAS 3.0 pharmacokinetic program. The maximum plasma concentration (C_*max*_) and time to reach the maximum concentrations (T_max_) were obtained directly from the curve.

### Results

#### Specificity

**Fig. 2:**
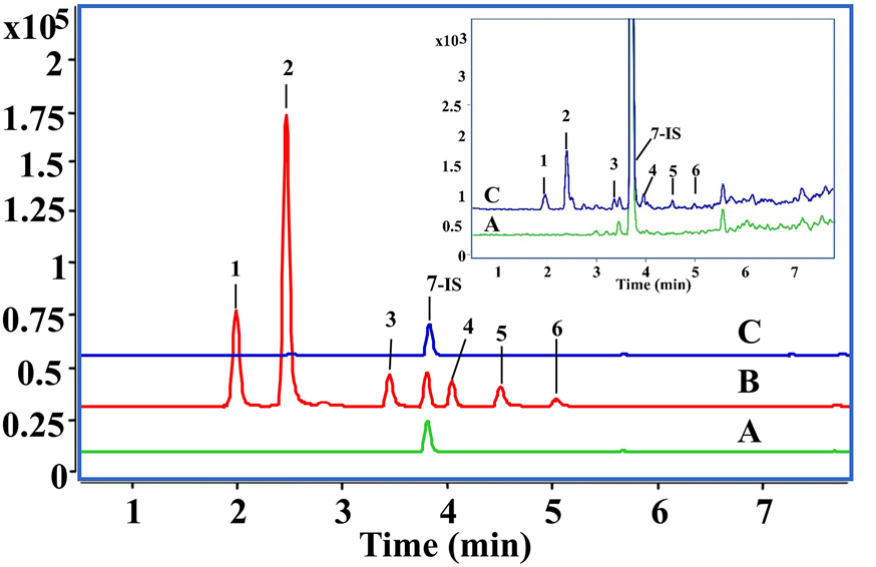
The total ion chromatograms of the active components and IS

**Fig. 3:**
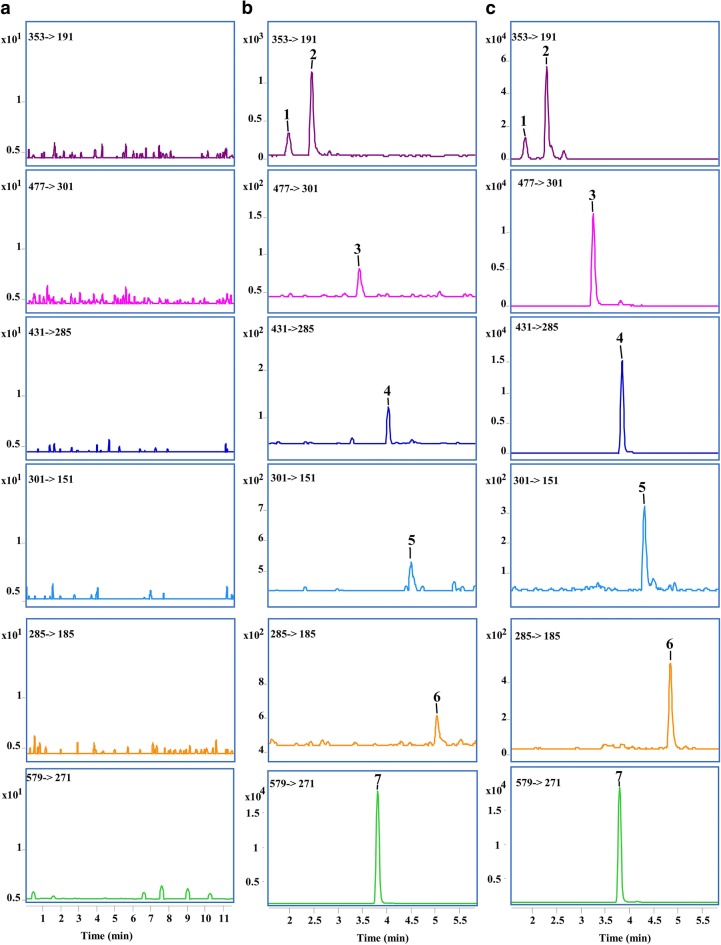
Typical MRM chromatograms of the six components in rats. **a** Blank plasma sample; **b** blank plasma samples spiked with standard mixtures and internal standards; and **c** rat plasma samples collected after oral administration of the CP extract within 30 min. Te: neochlorogenic acid (**1**), chlorogenic acid (**2**), quercetin-3-*O*-glucuronide (**3**), kaempferol-3-*O*-rhamnoside (**4**), quercetin (**5**), kaempferol (**6**), and naringin (IS **7**)

The total ion chromatograms of the active components and IS are depicted in Fig. 2. Typical MRM chromatograms obtained from blank plasma, blank plasma spiked with six analytes, and plasma samples from the rats after oral administration of CP are illustrated in Fig. 3. The retention time of neochlorogenic acid, chlorogenic acid, quercetin-3-*O*-glucuronide, kaempferol-3-*O*-rhamnoside, quercetin, kaempferol, and IS were 1.98, 2.46, 3.44, 4.05, 4.49, 5.02, 3.82 min, respectively. No interference was observed at the eluting times of either analytes or IS in blank plasma samples from rats, which indicated that the method exhibited good specificity.

#### Linearity and LLOQ

The equation of linear regression and linearity range for the six analytes are presented in Table 2. The results showed good linearity and all correlation coefficients were found to be higher than 0.9915. The LLOQ with a signal-to-noise (S/N) ratio of > 10 ranged from 0.78 to 15.63 ng/ml, which was sufficiently sensitive for our pharmacokinetic studies using rat plasma.

**Table 2:** The linear equation, linear range, and LLOQ of the six components in rat plasma samples

Analytes	Linear equation	Range (ng/ml)	R^2^	LLOQ (ng/ml)
Neochlorogenic acid	y = 44.588x − 394.53	7.32–7500	0.9998	7.32
Chlorogenic acid	y = 144.86x + 6.268	7.32–7500	0.9997	7.32
Quercetin-3-*O*-glucuronide	y = 48.271x − 2141.1	4.88–2000	0.9934	4.88
Kaempferol-3-*O*-rhamnoside	y = 264.73x + 764.38	0.98–500	0.9978	0.98
Quercetin	y = 114.8x − 32.66	0.78–100	0.9998	0.78
Kaempferol	y = 37.447x + 4.1182	15.63–250	0.9915	15.63

#### Precision and accuracy

The intraday and interday accuracy and precision data of five flavonoids in rat plasma are listed in Table 3. At each QC and LLOQ level, the interday and intraday precisions (RSD) of six active compounds ranged from 4.6 to 12.9% and the accuracy was − 13.2 to 15.1%. Both the intraday and interday results were found to be within the range of acceptance criteria.

**Table 3:** Precision, accuracy, extraction recovery, matrix effect of the six components in rat plasma samples

Analyte	Nominal concentration (ng/ml)	Intraday		Interday		Matrix effect (%, mean ± SD)	Extraction recovery (%, mean ± SD)
		Precision (RSD, %)	Accuracy (RE, %)	Precision (RSD, %)	Accuracy (RE, %)		
Neochlorogenic acid	120	12.9	− 11.3	11.2	− 10.8	110.5 ± 12.3	109.2 ± 13.5
	1200	11.8	9.3	9.9	12.5	102.2 ± 5.6	84.6 ± 12.9
	12,000	9.3	− 9.2	9.6	14.1	101.1 ± 12.1	80.5 ± 10.5
Chlorogenic acid	120	10.2	10.4	8.8	13.4	80.2 ± 12.7	106.8 ± 9.6
	1200	10.1	− 7.9	4.6	− 11.0	91.2 ± 15.3	90.1 ± 9.8
	12,000	9.5	10.9	9.2	9.6	99.4 ± 11.0	80.1 ± 13.4
Quercetin-3-*O*-glucuronide	40	12.5	13.0	5.8	− 12.6	115.1 ± 13.5	96.5 ± 12.6
	400	12.6	− 5.6	4.6	13.5	101.6 ± 12.9	116.3 ± 6.9
	4000	10.5	8.3	8.4	12.4	110.1 ± 12.4	115.4 ± 9.5
Kaempferol-3-*O*-rhamnoside	5	8.9	13.1	7.6	13.6	110.1 ± 6.3	93.0 ± 13.5
	50	8.2	15.1	4.8	4.9	115.6 ± 9.2	86.5 ± 14.8
	500	10.5	− 13.2	6.1	− 8.2	119.2 ± 9.4	94.4 ± 11.0
Quercetin	1.5	12.6	− 12.5	6.5	− 12.8	120.4 ± 11.3	89.5 ± 14.8
	15	10.9	13.6	8.3	10.4	119.5 ± 12.0	82.6 ± 13.9
	150	10.7	5.3	12.2	8.2	112.5 ± 10.1	89.0 ± 14.8
Kaempferol	20	6.8	12.5	5.4	− 12.5	83.5 ± 5.4	81.9 ± 11.8
	200	7.8	− 12.3	5.7	− 9.2	88.6 ± 9.9	86.6 ± 15.1
	2000	10.2	− 9.2	7.6	10.4	94.7 ± 8.4	83.5 ± 11.4

#### Extraction recovery and matrix effect

The data of extraction recovery and matrix effect of the six components are summarized in Table 3. The mean absolute recoveries of neochlorogenic acid, chlorogenic acid, quercetin-3-*O*-glucuronide, kaempferol-3-*O*-rhamnoside, quercetin, and kaempferol were 80.5 ± 10.5%–109.2 ± 13.5%, 80.1 ± 13.4%–106.8 ± 9.6%, 96.5 ± 12.6%–116.3 ± 6.9%, 86.5 ± 14.8%–94.4 ± 11.0%, 82.6 ± 13.9%–89.5 ± 14.8%, and 81.9 ± 11.8%–86.6 ± 15.1% at three QC levels. These results suggest that the sample preparation and extraction methods were stable and effective. The matrix effects derived from QC samples were between 80.2 ± 12.7% and 120.4 ± 11.3%. These results confirmed that there were no significant matrix effects.

#### Stability

The stability data of the six compounds in rat plasma, including post-preparation stability (storage for 24 h in the auto-sampler at room temperature), long-term stability (storage for 15 days at − 20 °C), and freeze–thaw stability (three freeze-and-thaw cycles at − 20 °C) are presented in Table 4. The results indicate that all analytes in rat plasma and processed samples under different storage conditions were stable.

**Table 4:** The stability of the six components in rat plasma samples

Analytes	Nominal concentration (ng/ml)	Frozen for 15 days at − 20 °C		Three-free-thaw cycles		Auto-sampler for 24 h	
		Precision (RSD, %)	Accuracy (RE, %)	Precision (RSD, %)	Accuracy (RE, %)	Precision (RSD, %)	Accuracy (RE, %)
Neochlorogenic acid	120	12.24	12.06	14.24	− 12.82	11.20	− 11.03
	1200	9.39	− 10.45	10.39	− 12.41	6.35	10.03
	12,000	9.36	7.35	10.36	5.35	9.33	13.02
Chlorogenic acid	120	12.03	− 9.60	14.03	− 15.01	13.36	12.53
	1200	10.02	8.03	11.63	14.06	12.59	− 9.36
	12,000	6.50	− 8.59	14.30	14.03	9.36	8.03
Quercetin-3-*O*-glucuronide	40	13.02	− 11.20	13.63	5.16	7.26	− 12.25
	400	8.20	14.06	10.96	− 11.52	5.88	4.62
	4000	9.56	10.12	15.05	8.29	9.26	− 9.13
Kaempferol-3-*O*-rhamnoside	5	14.01	12.58	9.86	− 9.86	3.89	− 9.59
	50	14.23	12.63	8.16	− 11.05	12.50	9.25
	500	15.20	15.03	12.03	7.16	6.23	− 6.33
Quercetin	1.5	10.36	− 12.3	7.59	7.68	9.51	14.59
	15	12.60	− 10.52	8.16	11.20	5.62	6.09
	150	10.24	9.35	12.14	− 15.01	8.45	11.13
Kaempferol	20	14.05	9.18	9.68	14.10	9.66	− 15.01
	200	10.02	− 11.05	15.11	− 15.02	7.33	11.06
	2000	9.05	11.56	9.13	11.30	6.03	11.42

#### Pharmacokinetic study

**Fig. 4:**
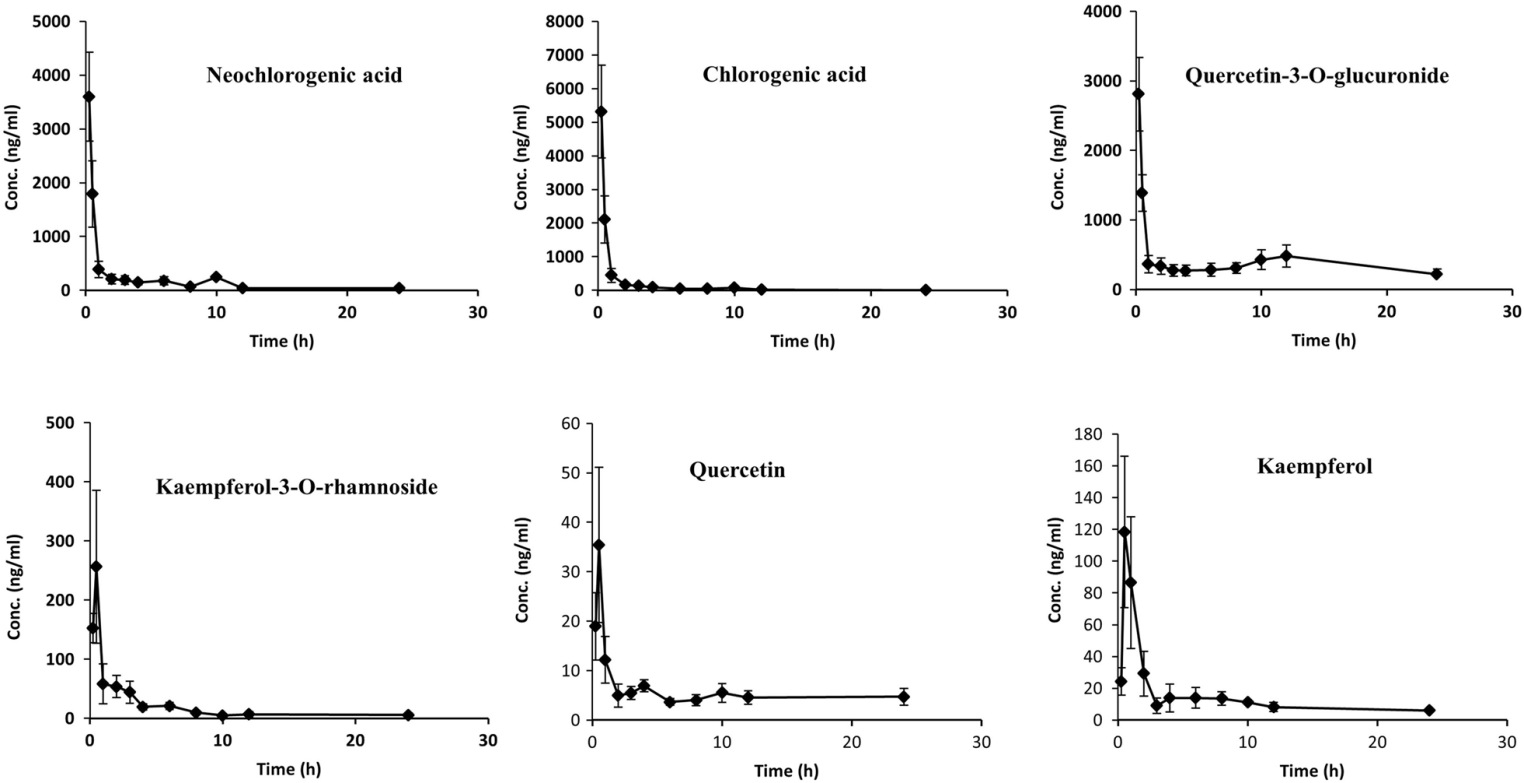
Mean plasma concentration–time curves of six analytes after oral administration of the CP extract

The present analytical method was employed to study the pharmacokinetics of neochlorogenic acid, chlorogenic acid, quercetin-3-*O*-glucuronide, kaempferol-3-*O*-rhamnoside, quercetin, and kaempferol in SD rats following oral administration of the CP extract at a dose of 10.5 g/kg body weight (corresponding to acute toxicity dosage). The mean plasma concentration–time profiles of the six analytes are illustrated in Fig. 3. The *T*_max_ values of all the analytes were within 1.0 h, thereby indicating fast absorption after oral administration of the CP extract. The pharmacokinetic parameters of six components were calculated by the DAS software using the non-compartmental model; the results are presented in Table 5. As shown in Table 5 and Fig. 4, the *C*_max_ of these six compounds ranged from 28.60 ± 8.23 to 4328.07 ± 1606.86 ng/ml because the content of six compounds varied greatly in the CP extract. Among these six analytes, two phenolic acids—neochlorogenic acid and chlorogenic acid—were found in the highest concentration (4328.07 ± 1606.86 ng/ml and 4169.13 ± 1888.68 ng/ml) in rat plasma postdosing.

**Table 5:** Main pharmacokinetic parameters of six analytes in rat plasma after oral administration of the CP extract

Analytes	Parameters				
	*C*_max_ (ng/ml)	*T*_max_ (h)	*t*_1/2_ (h)	*AUC*_0−t_ (µg/l*h)	*AUC*_0−∞_ (µg/l*h)
Neochlorogenic acid	4328.07 ± 1606.86	0.32 ± 0.12	4.54 ± 1.38	2453.18 ± 951.53	2471.38 ± 942.68
Chlorogenic acid	4169.13 ± 1888.68	0.39 ± 0.13	2.54 ± 0.53	3308.80 ± 1062.02	3578.30 ± 1203.93
Quercetin-3-*O*-glucuronide	3710.01 ± 1593.38	0.33 ± 0.13	10.04 ± 3.31	11,009.41 ± 2173.10	13,455.73 ± 1225.02
Kaempferol-3-*O*-rhamnoside	263.55 ± 119.77	0.42 ± 0.13	2.42 ± 1.29	553.16 ± 270.45	614.20 ± 254.97
Quercetin	28.60 ± 8.23	0.46 ± 0.10	11.70 ± 6.01	79.83 ± 28.49	91.68 ± 15.20
Kaempferol	121.82 ± 49.87	0.67 ± 0.26	7.24 ± 1.63	241.77 ± 76.98	324.73 ± 104.26

## Discussion

The contents of neochlorogenic acid and chlorogenic acid were a liter more than the flavonoids in the CP extract. This might be attributable to the hydrophilic group “COOH” of phenolic acids, which may facilitate the absorptions of phenolic acids, because phenolic acids can exist in a free form in plasma [20]. In addition, the maximum plasma concentrations of six compounds were achieved in a time frame from 0.32 to 0.67 h after oral administration, but their elimination half-life time (t_1/2_) varied greatly. Obviously, *T*_max_ values and t_1/2_ for neochlorogenic acid and chlorogenic acid were much lower than those for flavonoids—including quercetin-3-*O*-glucuronide, quercetin, and kaempferol—thereby indicating that phenolic acids are more easily metabolized than flavonoids owing to their transformation from neochlorogenic acid and chlorogenic acid to caffeic acid [21]. Moreover, biological activities of flavonoids ultimately depend on the systemic bioavailability of the aglycones and their metabolites in vivo. The t_1/2_ value of flavonoid glucoside kaempferol-3-*O*-rhamnoside was much lower than that of flavonoid glucoside quercetin-3-*O*-glucuronide, which may be attributed to the possible conversion of the flavonoids in the CP extract into quercetin-3-*O*-glucuronide by intestine microflora [22]. For example, quercetin-3-*O*-glucuronid could be formed from the hydroxyl carboxylation of isoquercitrin [23]. In addition, quercetin-3-*O*-glucuronide was the major circulating metabolite in the metabolism of flavonoids, which could be formed by a combination of quercetin and glucuronic acid in the liver [24]. It was probably easier for quercetin-3-*O*-glucuronide in the liver to secrete into bile than quercetin and then be drained into the intestines. Finally, quercetin could be released from quercetin-3-*O*-glucuronide through the hydrolysis of intestinal bacterial enzymes and be absorbed into the blood from the intestine, which implies that the concentrations of these two compounds could increase over time [25]. With regard to flavonoid aglycones, kaempherol may have a similar characteristic, thereby suggesting that this compound could also undergo an enterohepatic cycle. In addition, both quercetin and kaempherol have a much longer t_1/2_ value than that of flavonoid glucosides, kaempferol-3-*O*-rhamnoside, and quercetin-3-*O*-glucuronid. This is because both quercetin and kaempherol can be formed after the breakage of quercetin-3-*O*-glucuronide and kaempferol-3-*O*-rhamnoside or their other flavonoid glucosides in the CP extract, respectively [26].

## Conclusion

In this study, a UPLC–TQ/MS method for simultaneous determination of neochlorogenic acid, chlorogenic acid, quercetin-3-*O*-glucuronide, kaempferol-3-*O*-rhamnoside, quercetin, and kaempferol in rat plasma was developed and validated following oral administration of the CP extract. This method offered a better recovery, matrix effect, stability with a good precision, and accuracy and was successfully applied to a pharmacokinetic study of representative constituents from the CP extract. Therefore, the result obtained could provide useful information for future research on *Cyclocarya paliurus*.

## Data Availability

The data sets used during the current study are available from the corresponding author on reasonable request.

## Abbreviations

*Cyclocarya paliurus*: CP
UPLC–TQ-MS/MS: ultra-high-performance liquid chromatography coupled with a triple quadrupole electrospray tandem mass spectrometry
MRM: multiple reactions monitoring
IS: internal standard
ESI: electrospray ionization source
QC: quality control
LLOQ: linearity and lower limit of quantification
